# Encephalopathy caused by disulfiram

**DOI:** 10.1192/j.eurpsy.2021.1872

**Published:** 2021-08-13

**Authors:** R. Sant’Angelo, I. Piretti

**Affiliations:** 1 Mental Health Department, AUSL ROMAGNA, Cesena, Italy; 2 Psychologist, Hr Specialist, Independent Researcher, Cesena, Italy

**Keywords:** Encephalopathy, disulfiram

## Abstract

**Introduction:**

Disulfiram is an alcohol detox drug that has been approved by the FDA for over 50 years. Among the various side effects that can cause there is encephalopathy. Its incidence is currently unknown, according to some authors it is estimated between 1 and 20%.

**Objectives:**

In this article we report the case of a 48-year-old woman diagnosed with borderline personality disorder and alcohol use disorder, presenting with encephalopathy.

**Methods:**

We discuss about our diagnostic and therapeutic approach.

**Results:**

Fortunately, the rapid identification of this rare condition led to a favorable outcome in our patient.

**Conclusions:**

Early detection of any acute change in mental state, especially in early stage of therapy, is important. Cessation of disulfiram is recommended in case of suspicion about disulfiram encephalopathy. This case underscores the importance of awareness of this serious complication during disulfiram treatment. If suspected early, appropriate diagnosis and treatment can prevent rapid progression.

**Disclosure:**

No significant relationships.

